# The nature of gambling-related harm for adults with health and social care needs: an exploratory study of the views of key informants

**DOI:** 10.1017/S1463423619000549

**Published:** 2019-07-19

**Authors:** Stephanie Bramley, Caroline Norrie, Jill Manthorpe

**Affiliations:** 1 Research Associate, NIHR Health and Social Care Workforce Research Unit, The Policy Institute, King’s College London, London, UK; 2 Research Fellow, NIHR Health and Social Care Workforce Research Unit, The Policy Institute, King’s College London, London, UK; 3 Director, NIHR Health and Social Care Workforce Research Unit, The Policy Institute, King’s College London, London, UK

**Keywords:** **g**ambling, gambling-related harm, primary care, vulnerable adults

## Abstract

**Aim:**

To explore the views of professionals working within health, care and other agencies about harmful gambling among adults with health and social care needs.

**Background:**

Gambling is increasingly seen as a public health rather than an individual problem. Opportunities to gamble have grown in England in the last decade since the liberalisation of the gambling industry meaning that gambling is widely available, accessible and advertised within society. An estimated two million people in the UK are at risk of developing a gambling problem, some of whom may be adults with health and social care needs.

**Methods:**

Twenty-three key informants from primary care, social care services and third sector organisations in England were interviewed about their understanding of the risks to adults with health and social care needs from gambling participation.

**Findings:**

Thematic analysis revealed four themes: (1) gambling-related harm as a public health problem; (2) identification of groups of adults with health and social care needs who may be vulnerable to gambling-related harm; (3) factors potentially impeding the identification of gambling-related harm among adults with health and social care needs and subsequent help-seeking behaviour and (4) calls for professional development activities. Informants reported a perceived lack of awareness of gambling-related harm and a lack of a clear pathway or guidance which they could follow when supporting individuals experiencing gambling-related harm. Interviewees called for professional development activities to improve their knowledge and expertise in this area.

## Background

Debates about the widespread advertising of gambling products, the increased availability of fixed-odds betting terminals, rising participation in online gambling and the growing prevalence of problem gambling regularly feature in the UK media (MacInnes, 2017). Unsurprisingly, gambling-related harm has attracted more public health attention since the industry was deregulated in 2007. Furthermore, gambling-related harm is increasingly identified as a potential public health problem within leading medical and scientific communities (ie, Griffiths, S. 2017; The Lancet, 2017; Nature, 2018).

Gambling-related harm is defined as ‘the adverse impacts from gambling on the health and wellbeing of individuals, families, communities and society’ (Wardle *et al*., 2018). Currently, attention is being focused on identifying the types of gambling-related harm which people may experience, any associated risk factors and populations who may be particularly vulnerable to gambling-related harm (eg, Wardle, 2015; Langham *et al.*, 2016). The term ‘vulnerable adults’ is used for UK regulatory purposes, meaning ‘people who gamble more than they want to, people who may not be able to make informed or balanced decisions about gambling due to, for example, mental health problems, a learning disability or substance misuse relating to alcohol or drugs’ (Gambling Commission, 2016: 5.17).

Individuals working in primary care, social care services and third sector organisations come into contact with a wide range of individuals, some of whom may be experiencing gambling-related harm. For example, a survey of patients within 11 UK general practices found 0.9% exhibited problem gambling (Cowlishaw *et al.*, 2017). GPs working in Solihull, England reported seeing patients experiencing gambling-related harm, but many GPs had not received any training in relation to how to identify and treat gambling addiction (Chithiramohan and George, 2016). Therefore, the question of whether GPs, health and social care professionals working in the UK could or should do more to address problem gambling has been discussed, with low levels of awareness of problem gambling being identified as potential barriers to practitioners becoming involved in the identification and management of problem gambling (Sanju and Gerada, 2011; Bramley *et al.*, 2019).

The English Local Government Association (2013) observed that gambling problems affect the health and well-being of local communities and wider society, and urged local government, with partners, to develop a coherent approach to problem gambling, with a focus on preventative work with high-risk groups. It recommended that local health agencies should raise awareness of problem gambling among primary care professionals and work with local government to direct people to local and national support services. It advised mental health service providers to consider how to better identify problem gambling and provide access to specialist support. Local audit, clinical and public research and evaluation of interventions across health and social care partnerships were described as having the potential to support the national evidence base and develop the ‘business case’ for intervention. Licensing, planning, trading standards and local government scrutiny processes were identified as needing to bring together public bodies and gambling operators to establish the nature and extent of local problems.

Recent scoping reviews (Bramley *et al.*, 2017, 2018) have noted the lack of evidence about the nature and impact of gambling-related harm on adults with health and social care needs and prompted this present study. Service-related data about gambling-related harms do not generally distinguish those affected by health or care needs from the general population, although estimates of the extent of gambling behaviour have been calculated in respect of some groups, for example, homeless populations (Sharman *et al*., 2014). Furthermore, evidence of a ‘harm paradox’ has been obtained for migrant populations suggesting that migrants may be less likely to gamble but more likely to experience gambling-related harm (Wardle *et al.*, 2019).

## Aim

The aim of this study was to explore the views of key informants working within health, social care and other agencies about harmful gambling among adults with health and social care needs.

## Method

### Sample and recruitment

We devised a sampling framework to provide a broad range of interviewees and sought volunteer participants from different services and organisations. We explained that the study was explorative and that no patient or service user data would be sought or identifiable data reported. Informed consent was obtained before interviews commenced. Semi-structured telephone or face-to-face interviews were conducted between September 2016 and May 2017. Twenty-three key informants (14 male and 9 female) involved in the care and support of adults with health and social care needs (including medical and care professionals) from National Health Service (NHS), local government, charities or third sector organisations and gambling experts were interviewed (see Table 1).

**Table 1.** Interviewees

Interviewee	Gender
GP1 (older people)	Male
GP2 (mental health)	Female
Counselling Services Manager	Male
Social Work Lecturer	Male
Psychiatrist	Female
Dementia Nurse	Female
Frontline Services Manager	Male
Gambling Charity Employee 1	Male
Trainer (Vulnerable adults and older people)	Male
Occupational Therapist	Male
Head Injury Charity Worker	Female
Psychiatrist (Parkinson’s Disease Specialist)	Male
Social Care Addictions Specialist	Female
Gambling Charity Employee 2	Male
Autism Charity Employee	Male
Debt Management Charity Employee	Female
Older People’s Charity Employee	Male
Solicitor	Male
Elderly People Charity Employee	Male
Betting Shop Employee	Male
Money Advice Charity Employee	Female
Homeless Charity Employee	Female
Nurse Co-ordinator (Parkinson’s Disease Charity)	Female

### Materials

The interview schedule consisted of open-ended questions designed to capture key informants’ experiences of working with people with health and social care needs who had experienced gambling-related harm (Appendix 1). Interviews were audio recorded, with consent, and transcribed.

### Data analysis

Transcripts were inputted into NVivo7 to facilitate data analysis. Data were analysed using thematic analysis which enabled the authors to scrutinise data in detail through identifying, analysing and reporting themes (patterns) within data (Braun and Clarke, 2006). We followed the five phases of thematic analysis – (1) familiarisation with the data (the research team repeatedly read the transcripts); (2) generating initial codes; (3) searching for themes; (4) reviewing themes and (5) defining and naming themes (Braun and Clarke, 2006).

### Findings

Four main themes were identified in the data: (1) gambling as a public health problem; (2) identification of groups of adults with health and social care needs who may be vulnerable to gambling-related harm; (3) factors potentially impeding the identification of gambling-related harm among adults with health and care needs and subsequent help-seeking behaviour and (4) call for professional development activities.

#### Gambling-related harm as a public health problem

Many interviewees considered gambling-related harm as a public health problem and identified ways that gambling had negatively impacted on adults with health and social care needs. One interviewee reported gambling participation initially as a distraction for patients but became a habit and ultimately an addiction or provoked high anxiety. The potential consequences of gambling included financial difficulties leading to other problems including depression, with one interviewee commenting ‘and then they not only have to get help for their addiction, but also how they’re going to cope with their money difficulties as well’ (Trainer).

Interviewees also reported challenges when supporting adults with health and social care needs experiencing gambling-related harm. A gambling support charity had been supporting a man with several mental health problems but, when his inheritance ran out, he gambled with his benefit payments. The charity liaised with his housing provider and other charities to build a routine for him, but the man refused to engage with mental health services and consequently his mental health was negatively affected.

However, unlike many other public health concerns, gambling participation was not always viewed negatively. Gambling participation was seen as a positive activity helping people be active, be social and engage in activities which they participated in prior to illness. An Occupational Therapist specialising in mental health and older people provided an example of where a client’s family supported his gambling because ‘he used to go to the betting shop…. it got him out of the house, he would see people in the betting shop, which would make it into a social activity’.

Nevertheless, interviewees argued that the responsibility for addressing gambling-related harm should be shared by industry, government, the regulator of gambling and local authorities. However, because the vast majority of funding for UK-specific gambling support services comes from voluntary donations from the gambling industry, one interviewee thought that government should seek to increase industry contributions (Gambling Charity Employee 2).

Others called for a national strategy to tackle gambling-related harm, as exists for substance misuse:

–“I don’t think it’s any different to alcohol or cigarette addition… if we deal with those in the NHS…. then why shouldn’t we deal with gambling addiction too?”. (GP1, mental health)

#### Identification of groups of adults with health and social care needs who may be vulnerable to gambling-related harm

Interviewees were asked to identify groups they thought might be vulnerable to gambling-related harm. Broad descriptions were obtained, some of which were circular, ‘anybody with gambling-related harm is vulnerable’ (Gambling Charity Employee 2). The same interviewee thought that there were risks to almost everyone, ‘there’s always potential for that harm to escalate; there’s always potential for that harm to cause other harms’.

Some interviewees listed specific medical conditions they thought may be associated with gambling-related harm. Examples featuring people with mental health problems or dementia were provided by several interviewees. One clinician considered that people with:

“… no confidence, no self-esteem…a person who might have learning disabilities, somebody who is perhaps demented…people with post-traumatic stress disorder…, people with eating disorder, people with substance misuse and dependence might experience gambling-related harm”. (Psychiatrist)

Another thought that people living with schizophrenia or bipolar disorder could experience gambling-related harm (Gambling Charity Employee 2).

Older people might also be vulnerable because of changes in circumstances, ‘bereavement, loss of employment through retirement … loss of their status in society … feeling depressed because they’re lonely, isolated’ (Psychiatrist). An Older People’s Charity Employee summarised the appeal of betting shops for older people – ‘if they go into a betting shop and get a smiling face from someone, and they don’t see anyone else, then they might well go back because they’re going to get a smiling face and maybe a few coffees as well’.

The appeal of gambling environments as places of safe social interaction emerged within another example from Gambling Charity Employee 2 who had been asked by the local NHS mental health service to discourage a patient from spending his benefits in betting shops. However, ‘it became quite clear … actually, this was such a key part of his life, we couldn’t just say stop going … that’s not going to work; we would have to do a more prolonged piece of work where we’re offering alternatives to him’. Being in the betting shop alleviated anxieties: ‘he was quite stressed, quite anxious and for him to be going to an environment where they’re very friendly towards him, give him tea and coffee, etcetera, that was a very powerful experience for him and it wasn’t one he was ready to give up’.

Further specific examples concerning people with learning disabilities were provided. A Trainer provided information about a young man with learning disabilities who plays bingo: ‘every time I see him he always tells me about what he’s won; never tells me what he’s lost, and he really doesn’t see the risk’. The interviewee considered his parents encouraged gambling because ‘he has challenging behaviour, and they quite like it when he’s not around. They want some time to themselves’ (Trainer). However, they predicted ‘it won’t be long, I believe, before he starts … getting into more serious gambling, visiting casinos’. Concerns were growing about his use of benefit payments for gambling.

There were accounts of people with learning disabilities being supported by gambling venue staff – ‘they were sort of looked after almost by some of the staff, they would make sure they weren’t getting fleeced or taken advantage of by maybe not understanding something’ (Betting Shop Employee). Such customer care was reportedly undertaken without guidance from their employer. It had extended in one case to escorting a customer home, so that he would not be subjected to verbal or physical abuse by local children (Betting Shop Employee). However, a small number of interviewees commented on individuals who had ‘ended up blowing a huge amount of money in their local betting shop’ without any intervention from staff (Social Work Lecturer).

People with mobility problems were also considered to be vulnerable to gambling-related harm. A Counselling Services Manager recalled a wheelchair-using client who seemed to be using online gambling as a coping mechanism.

An Autism Charity Employee commented on the potential dangers of gambling for people with autism; in their view, avoidance of asking for help is characteristic of the condition:

“someone could spend hours in a betting shop analysing things and reading the stuff on the walls … it can … really draw people in, and so if you’ve got that kind of autistic mind-set, then I wouldn’t be surprised if a lot of the people spending all day in a betting shop, some of them are on the spectrum”. (Autism Charity Employee)

People experiencing homelessness were thought to be vulnerable as they could get ‘a free drink and a bit of warmth’ in high street betting shops (Gambling Charity Employee 1). Such environments may also be perceived as ‘a different space to be in… a space that’s less judgemental and visible than some other public places’ (Homeless Charity Employee). Homeless people may gamble in order to do ‘something they can control and make decisions about’ and perceive betting shops as ‘a constant, familiar place that you can access and feel the familiarity and somewhere you would feel relaxed, or at home’ (Homeless Charity Employee).

People who lack mental capacity or live with cognitive impairment were mentioned as potentially vulnerable to gambling-related harm as they may not have ‘an understanding of the implications of it for them now and in the future’ (Dementia Nurse). This view was echoed by another interviewee who remarked that some individuals may continue to gamble but ‘don’t really understand what it is they’re doing’ and ‘may not be aware of the eventual outcome’ (ie, losing money and addiction) (Psychiatrist).

Certain prescribed medications were also identified as a factor which may contribute to gambling-related harm. GP1 (with expertise in mental health) considered that selective serotonin reuptake inhibitors (SSRIs) were ‘associated with increased risk-taking in vulnerable people’. They also reported that dopamine agonist drugs, which may be prescribed to people diagnosed with Parkinson’s Disease were associated with increased risk-taking but thought it would be:

“… pretty unusual to use those in the same group where you’re concerned about gambling. I don’t think that’s a massive contribution factor to people’s gambling problems, because … they’re mainly used in Parkinson’s and …so you’re mainly talking about people in their 80s and 90s who are far less likely to be gambling”.

However, the Frontline Services Manager for a gambling charity (not clinically trained) reported that ‘it’s a documented fact that some of the medication associated with Parkinson’s Disease can lead to a reduction in inhibition’ and said they had received calls ‘from partners, children, where they’re concerned about an elderly relative’. Furthermore, dopamine agonist drugs were referred to by a Parkinson’s Disease Nurse (working in the community) who recalled the case of a patient who had never gambled but then spent thousands of pounds on online gambling activities:

“She was saying, information is so readily available, on the TV, on the internet and she could even use a Kindle (hand held computer) as well as a phone, and she just couldn’t resist that urge, and was completely overwhelmed by it”.

This interviewee also reported cases where spouses had telephoned their service to seek help for a relative who was taking dopamine agonist drugs and ‘spending huge amounts of money’ (Parkinson’s Disease Nurse).

#### Factors potentially impeding the identification of gambling-related harm among adults with health and social care needs and subsequent help-seeking behaviour

Within the interviews, many identified a number of factors which may potentially impede the identification of gambling-related harm among adults with health and social care needs and individuals who experience gambling-related harm engaging in help-seeking behaviour.

Interviewees identified that there was no pathway or guidance to follow in relation to the diagnosis, assessment and management of gambling within primary care, which differed from other addictive behaviours such as smoking, alcohol and drug misuse. Therefore, professionals working in primary care did not screen for gambling-related harm. Gambling was described as an ‘under-detected problem’ by one GP (GP2, mental health). Another GP reported that he did not ask about gambling problems during consultations because the NHS does not ‘have an answer to it, so we don’t look for it’ and because ‘a lot of medicine is based on having a solution to a problem, if you have the solution, you can see the problem. If you don’t have a solution, then you tend not to want to see the problem’. A Psychiatrist considered that the identification of problem gamblers would ‘very much depend on people presenting themselves with a problem, rather than it being identified through a systematic method’.

Other interviewees acknowledged that the lack of visible signs of gambling-related harm compared with the signs of alcohol or drug misuse may contribute to professionals being unaware that adults with health and social care needs may be experiencing gambling-related harm. A Counselling Services Manager compared the signs of alcohol and drug addiction to gambling – ‘you don’t see gambling addiction sitting in front of you … there’s no physical, …direct physical consequences, where there would be with someone who’s a heroin addict or an alcoholic … it’s less visible’. He added that while there ‘are a lot of tell-tale signs in terms of behaviours, but at the same time, a lot of problem gamblers are able to conceal’. Therefore, the family members of adults with health and social care needs experiencing gambling-related harm may also be unaware that gambling is impacting upon individuals. For example, gamblers may ‘apprehend the mail … make sure no one sees their laptop or tablet or phone’ in order to hide their gambling.

Professionals may also only encounter instances of gambling-related harm after a prolonged period of time or when an individual is no longer in receipt of a service. A homeless charity discovered, by chance, the extent of a client’s gambling participation ‘when we were clearing each room (in an abandoned property) and found all the gambling receipts’.

Interviewees’ responses indicated that the onus was placed on adults with health and social care needs to disclose any gambling problems. However, such disclosures may be unlikely to occur if individuals are living with a number of medical conditions because gambling problems may not be ‘top of the agenda’ (Gambling Charity Employee 2). This view was supported by another interviewee:

“one particular client that we were aware of gambling regularly had suffered a brain injury, was a dependent drinker and was occasionally using drugs and may or may not have had other mental health issues that hadn’t been diagnosed because the brain injury and alcohol addiction were the presenting issues, so we hadn’t been able to do further work”. (Homeless Charity Employee)

Several interviewees also identified factors which may impede adults with care and support needs engaging in help-seeking behaviours. Inaccessibility was one reason, as services are not widely available or known about (Counselling Services Manager). Other barriers included costs associated with travelling to the treatment provider and service limitations (Counselling Services Manager). Also mentioned were poor communication skills, feeling ashamed or embarrassed, fear of losing welfare benefit payments (if they use such payments to fund gambling), difficulty keeping appointments and problems such as depression affecting help-seeking.

The provision of services available to those experiencing gambling-related harm was also thought to potentially impact upon help-seeking behaviour by adults with health and social care needs. Waiting lists for NHS-funded services which may be able to support those experiencing gambling-related harm were perceived to be too long. Furthermore, the priority given to gambling problems was thought to differ between NHS-funded services and private-funded services. Private services tend to recognise gambling ‘as a primary addiction requiring rehab(ilitation) and on-going support’, whereas the NHS tended to prioritise the treatment of other co-morbid conditions or treat the addiction and not the other condition (Trainer). Calls for gambling-related harm to be better tacked by the NHS were made by several participants.

#### Call for professional development activities

Most interviewees, apart from those working within gambling support services, were generally unaware of the types of support available to adults with health and social care needs experiencing gambling-related harm. Therefore, treatment options were not generally discussed by interviewees. Exceptionally, one interviewee outlined the case where a patient had unsuccessfully completed a year’s course of Cognitive Behavioural Therapy because she could not process or retain information. The patient was still spending her benefit money in betting shops and begging in public. Although this patient would never ‘reach rock bottom because she’s in supported housing’, the support workers called for professional development activities which focused on being able to identify ‘red flags’ or triggers for problematic gambling.

There was little awareness of gambling management tools to which practitioners could signpost adults with health and social care needs so as to help them to control their gambling participation. Such tools include setting time and monetary limits when gambling, self-exclusion schemes (which enable individuals to bar themselves from gambling environments and online gambling websites for a set period of time) and software, which prevent individuals from accessing online gambling websites.

One interviewee reported that it might be beneficial for practitioners if there was more partnership working and links formed with specialist gambling services. For example, a charity had forged a partnership with a gambling support charity, and this was perceived to help them feel equipped to support adults with health and social care needs who were at risk of experiencing gambling-related harm. Another interviewee suggested that gambling operators could alert a professional if ‘they’re worried about somebody’ (GP2, mental health). This view was shared by another interviewee who thought that forming better relationships between gambling operators and support agencies would help both parties gain ‘valuable advice and guidance’ (Trainer). Another considered that ‘working in a much more co-productive way’ would facilitate sharing of expertise, skills and knowledge (Gambling Charity Employee 1).

Some interviewees thought that pathways and guidance needed to be developed, so that practitioners could signpost adults with health and social care needs to support services and encourage individuals to engage in help-seeking behaviours. Several also thought that it was important for practitioners to receive training via professional development activities so as to improve their knowledge of gambling-related harm. Furthermore, some thought that information about gambling and gambling support services should be developed for dissemination by practitioners to adults with care and support needs. However, it would be important for this information to be accessible – ‘Some of the information that is given out about the consequences of gambling may not be easily understood by somebody with cognitive difficulties, somebody that is not good with words … so …. much more pictorial information …. available in a number of formats’ (Trainer).

## Discussion

This interview study explored practitioners’ knowledge about gambling-related harm among adults with health and social care needs. Representatives from organisations working with adults with care and support needs drew on their experiences to discuss services and possible public health measures to address gambling-related harm, echoing calls made by Bowden-Jones (2018), Griffiths, S. (2017), Griffiths, M. (2017) and the Responsible Gambling Strategy Board (2016).

Some interviewees considered gambling-related harm as a public health issue and called for it to be recognised as such. This was because gambling was thought to impact some individuals’ mental health, financial situation, housing situation and relationships. In addition, several identified that loneliness, feeling unsafe, being isolated, taking SSRIs or dopamine agonists could be risk factors for adults with health and social care needs experiencing gambling-related harm. However, several interviewees acknowledged that gambling could be a positive activity for some individuals.

Ideas of who might be vulnerable to gambling-related harm ranged from a broad definition (ie, anyone) to those with specific health conditions (eg, cognitive impairment) to specific population groups (eg, people experiencing homelessness; older people). Such ideas emerged from experiences of clients/patients who seemed to be experiencing gambling-related harm. This knowledge could be used to screen certain population groups, target health campaigns and direct practitioners’ efforts to help affected individuals to manage their gambling participation.

Apart from those who worked for gambling charities, no other interviewees discussed gambling during consultations or screened for gambling problems during initial assessments. There were strong feelings that people did not disclose their own or others’ gambling problems, and therefore, indices of gambling-related harm were sometimes only discovered by chance. These experiences highlight possible opportunities for those involved in the support of adults with care and support needs to be more aware of gambling as a potentially problematic behaviour, trained so that they can have open and probing conversations with their clients/patients and their carers or supporters, and be equipped with knowledge of where to signpost affected individuals to treatment, counselling or safeguarding services. There may be opportunities for more professionals working within primary care, including GPs, nurses, pharmacists and receptionists to be involved with signposting, making referrals and providing affected individuals with a space to talk about their gambling problems.

Overall, there was an underlying theme that none of the organisations could address these risks alone and partnerships were needed between organisations including the NHS, social services, housing and care providers, and the gambling industry in order to minimise the risk of adults with care and support needs experiencing gambling-related harm. Such partnerships may help improve care for those experiencing gambling-related harm as practitioners’ knowledge about gambling-related harm should improve, practitioners may feel better equipped to identify affected individuals and refer people to support services.

The interviews provided insights into what these largely non-specialist (in terms of gambling) key informants know about harmful gambling among adults with health and social care needs. However, limitations should be borne in mind. First, our sample was purposefully recruited and only 23 interviews were conducted. Larger studies would help determine the views of others who support adults with health and social care needs. Second, interviewees may have recalled cases that caused them concern, only partially recalled cases, recalled exceptional cases and/or those which occurred some time ago and so may be subject to risks of hindsight; we did not ask them to review case notes or similar. Nonetheless, this study provides a springboard for other research and its contemporary nature highlights the increasing practices of gambling online and by phone, with their consequent invisibility.

## Conclusion

Those working across a range of health and social care agencies, third sector, charity and other organisations report encountering cases of gambling-related harm among adults with care and support needs. Interviewees highlighted a need for pathways and guidance to be developed, together with professional development activities to improve awareness of gambling-related harm and professionals’ ability to support affected individuals.

## Appendix 1: Interview schedule

1. Please could you tell me about your background and professional experience.
2. What experience have you had in dealing with adults with health and care needs who have experienced gambling-related harm?
3. What is current practice in order to manage adults with health and care needs experiencing gambling-related harm?
4. In your view are there any factors which may be linked to adults with health and care needs experiencing gambling-related harm?
5. To what extent do you feel equipped to deal with issues related to gambling-related harm experienced by adults with health and care needs?
6. To what extent do you consider that gambling-related harm is a public health issue?
7. In your opinion are there any factors which may impact on the help-seeking behaviour of adults with health and care needs?
8. How might such barriers be overcome?
9. What things would you like to see or be implemented in the future in order to minimise the extent that adults with health and care needs experience gambling-related harm?
